# Novel DNA Methylation Sites Influence *GPR15* Expression in Relation to Smoking

**DOI:** 10.3390/biom8030074

**Published:** 2018-08-20

**Authors:** Tina Haase, Christian Müller, Julia Krause, Caroline Röthemeier, Justus Stenzig, Sonja Kunze, Melanie Waldenberger, Thomas Münzel, Norbert Pfeiffer, Philipp S. Wild, Matthias Michal, Federico Marini, Mahir Karakas, Karl J. Lackner, Stefan Blankenberg, Tanja Zeller

**Affiliations:** 1 Clinic for General and Interventional Cardiology, University Heart Center Hamburg, 20246 Hamburg, Germany; t.haase@uke.de (T.H.); ch.mueller@uke.de (C.M.); j.krause@uke.de (J.K.); roethemeierC@rki.de (C.R.); m.karakas@uke.de (M.K.); s.blankenberg@uke.de (S.B.); 2 German Centre for Cardiovascular Research (DZHK), 13316 Berlin, Germany; j.stenzig@uke.de (J.S.); waldenberger@helmholtz-muenchen.de (M.W.); tmuenzel@uni-mainz.de (T.M.); philipp.wild@unimedizin-mainz.de (P.S.W.); Karl.Lackner@unimedizin-mainz.de (K.J.L.); 3 Institute of Experimental Pharmacology and Toxicology, University Medical Center Hamburg-Eppendorf (UKE), 20246 Hamburg, Germany; 4 Research Unit of Molecular Epidemiology, Helmholtz Zentrum München, German Research Center for Environmental Health, 85764 Neuherberg, Germany; sonja.kunze@helmholtz-muenchen.de; 5 Institute of Epidemiology II, Helmholtz Zentrum München, German Research Center for Environmental Health, 85764 Neuherberg, Germany; 6 Center for Cardiology, Cardiology I, University Medical Center Mainz, Johannes Gutenberg University-Mainz, 55131 Mainz, Germany; 7 Center for Thrombosis and Hemostasis, University Medical Center Mainz, Johannes Gutenberg-University Mainz, 55131 Mainz, Germany; 8 Center for Translational Vascular Biology (CTVB), University Medical Center Mainz, Johannes Gutenberg-University Mainz, 55131 Mainz, Germany; 9 Department of Ophthalmology, University Medical Center of the Johannes Gutenberg-University Mainz, 55131 Mainz, Germany; norbert.pfeiffer@unimedizin-mainz.de; 10 Preventive Cardiology and Preventive Medicine, Center for Cardiology, University Medical Center of the Johannes Gutenberg-University Mainz, 55131 Mainz, Germany; 11 Center for Thrombosis and Hemostasis, University Medical Center of the Johannes Gutenberg-University Mainz, 55131 Mainz, Germany; 12 Department of Psychosomatic Medicine and Psychotherapy, University Medical Center of the Johannes Gutenberg-University Mainz, 55131 Mainz, Germany; Matthias.Michal@unimedizin-mainz.de; 13 University Medical Center, Institute of Medical Biostatistics, Epidemiology and Informatics (IMBEI), 55131 Mainz, Germany; marinif@uni-mainz.de; 14 Institute of Clinical Chemistry and Laboratory Medicine, University Medical Center, Johannes Gutenberg University Mainz, 55131 Mainz, Germany

**Keywords:** GPR15, smoking, biomarker, DNA methylation

## Abstract

Smoking is a major risk factor for cardiovascular diseases and has been implicated in the regulation of the G protein-coupled receptor 15 (GPR15) by affecting CpG methylation. The G protein-coupled receptor 15 is involved in angiogenesis and inflammation. An effect on *GPR15* gene regulation has been shown for the CpG site CpG3.98251294. We aimed to analyze the effect of smoking on *GPR15* expression and methylation sites spanning the *GPR15* locus. DNA methylation of nine *GPR15* CpG sites was measured in leukocytes from 1291 population-based individuals using the EpiTYPER. Monocytic *GPR15* expression was measured by qPCR at baseline and five-years follow up. *GPR15* gene expression was upregulated in smokers (beta (ß) = −2.699, *p*-value (*p*) = 1.02 × 10^−77^) and strongly correlated with smoking exposure (ß = −0.063, *p* = 2.95 × 10^−34^). Smoking cessation within five years reduced *GPR15* expression about 19% (*p* = 9.65 × 10^−5^) with decreasing *GPR15* expression over time (ß = 0.031, *p* = 3.81 × 10^−6^). Additionally, three novel CpG sites within *GPR15* affected by smoking were identified. For CpG3.98251047, DNA methylation increased steadily after smoking cessation (ß = 0.123, *p* = 1.67 × 10^−3^) and strongly correlated with changes in *GPR15* expression (ß = 0.036, *p* = 4.86 × 10^−5^). Three novel *GPR15* CpG sites were identified in relation to smoking and *GPR15* expression. Our results provide novel insights in the regulation of GPR15, which possibly linked smoking to inflammation and disease progression.

## 1. Introduction

Cigarette smoking severely increases the risk for life-threatening diseases such as cardiovascular diseases, cancer, and chronic respiratory diseases with over 7 million deaths attributed to tobacco worldwide [1]. Cigarette smoke contains more than 5000 compounds with many of them, upon entering the blood stream, negatively affecting organs and cells [2]. However, the exact molecular mechanisms showing how tobacco substances influence disease onset and progression remain elusive.

Within the last several years, the field of epigenetics has emerged and has provided new approaches and insights into the regulation of gene expression. It was shown that the modification of a cytosine followed by a guanine nucleotide (CpG) to 5-methylcytosine in the gene promoter region may result in a decrease of transcriptional activity of the corresponding gene [3]. Several studies have been published by focusing on the effect of cigarette smoke on methylation and changes in methylation status in response to smoking have been shown for several genes such as *AHRR* and *F2RL3* [4,5]. These genes have been implicated in cell proliferation, immune response, and detoxification, which can contribute to the pathogenesis of smoking-associated diseases [6].

The CpG site CpG3.98251294 was identified in relation to smoking within the *GPR15* gene, which encodes the G protein-coupled receptor 15 [7,8,9]. Smoking was associated with decreased CpG3.98251294 methylation and increased G protein-coupled receptor 15 gene *(GPR15)* expression [8,9,10,11]. This effect was slowly reversible after smoking cessation [7,11,12]. Additionally, CpG3.98251294 methylation correlated with increased cumulative smoking exposure [13]. The transmembrane receptor GPR15 is mainly expressed on T cells and acts as a coreceptor for human immunodeficiency virus and simian immunodeficiency virus [14,15,16]. The G protein-coupled receptor 15 has also been shown to be involved in angiogenesis and inflammation [17,18,19,20,21,22], which indicated a role of GPR15 in smoking-associated diseases such as cardiovascular diseases and cancer. Hence, *GPR15* poses an interesting new candidate gene of smoking-related diseases and smoking-induced molecular pathways. However, to date, the influence of smoking on only one CpG site (CpG3.98251294) within the *GPR15* gene region has been described [7,8,9,10,11,13].

The aim of the present study was to analyze the association between smoking, *GPR15* methylation and expression and to gain further knowledge of additional DNA methylation sites spanning the *GPR15* locus. This study identified three novel DNA methylation sites within the *GPR15* locus related to smoking as well as *GPR15* expression. 

## 2. Materials and Methods 

### 2.1. Study Participants

In this study, 1291 individuals in the population-based Gutenberg Health Study were analyzed [23]. RNA and DNA were collected, as described previously [24]. Self-reported smoking statuses were classified as follows: current smokers (including occasional smokers), ex smokers (smoking cessation at least six weeks before study participation), and never smokers. Cumulative smoking exposure was evaluated by pack years (one pack year = smoking of 20 cigarettes per day for one year) for current smokers and ex smokers. For longitudinal gene expression (follow up), participants visited the same study center five years after baseline recruitment. Written informed consent was obtained from all study participants. The study protocols and sampling design were approved by the local ethics committee of the Medical Chamber of Rhineland-Palatinate, Germany (ethical approval code 837.020.07 (5555)).

### 2.2. RNA and DNA Isolation

Monocytic RNA was isolated as described previously [24]. Blood was collected using the Vacutainer CPT Cell Preparation Tube System (BD Biosciences, San Jose, CA, USA) and monocytes were enriched by negative selection with the Rosette Sep Monocyte Enrichment Cocktail (StemCell Technologies, Vancouver, BC, Canada), which leads to 72% to 85% of enriched monocytes. Total RNA was isolated using Trizol extraction and purification by silica-based columns. Genomic DNA from leukocytes was extracted as described by Miller et al. from buffy-coated ethylenediamine tetraacetic acid blood samples [24,25].

### 2.3. Analysis of GPR15 Gene Expression

Monocytic *GPR15* mRNA expression was measured by real-time quantitative polymerase chain reaction (qPCR) using the 7900 TaqMan system (Applied Biosystems, Vancouver, BC, Canada). RNA was reverse transcribed using High-Capacity cDNA Reverse Transcription Kit (Applied Biosystems), according to manufacturers’ protocols. For *GPR15* gene expression analyses, the *GPR15* Hs00922903_s1 gene expression assay was used (Applied Biosystems). Quantification of the housekeeping gene *GAPDH* (Hs99999905_m1, Applied Biosystems) as an internal control was performed for each sample. Expression of *GPR15* mRNA was normalized to *GAPDH* mRNA expression by calculating ΔCt values.

### 2.4. Analysis of DNA Methylation 

Analysis of *GPR15* DNA methylation was carried out with the EpiTYPER MassARRAY technology (Agena Bioscience, San Diego, CA, USA), according to the manufacturer’s protocols, which allowed region-specific quantification of CpG sites [26]. Genomic DNA was bisulfite modified to convert un-methylated cytosines into uracils. One μg of DNA was treated with sodium bisulfite using the EZ methylation kit (Zymo Research, Irvine, CA, USA), according to the manufacturers’ protocols. Ten ng of bisulfite DNA was amplified by polymerase chain reaction (PCR) using the following primers: 5′-aggaagagagGTTTTTTGGTGATGGATTTAGAAGA-3′ and 5′-cagtaatacgactcactatagggag aaggctTAAACAAAAAAATAAACAACCCCAA-3′, which results in a 523 bp-fragment. Subsequently, DNA was reverse transcribed, fragmented, and analyzed by mass spectrometry. The *GPR15* gene includes 15 CpG sites. Nine of these CpG sites (CpG3.98250924 to CpG3.98251294) were located within the 523 bp-fragment and were labeled, according to Saffery et al. [27]. Six of these CpG sites could be analyzed. Due to the low mass of the cleavage product, which leads to unreliable methylation values, CpG3.98250924 and CpG3.98251081 could not be covered by the assay. CpG3.98251268 was not included in the analyses since it was fully methylated in 90% of the samples. CpG3.98251294 corresponds to the previously described cg19859270 site. The *GPR15* locus including the CpG sites CpG3.98250924 to CpG3.98251294 is depicted in Figure S1. More detailed information on the mass fragments detected by the EpiTYPER are given in Table S1.

### 2.5. Statistical Analysis

Associations between cigarette smoking status, *GPR15* mRNA expression, and *GPR15* DNA methylation were calculated by linear mixed regression models and adjusted for age and sex. Changes in *GPR15* gene expression between the baseline and a five-years follow up visit were analyzed by a paired *t*-test. The threshold for statistical significance was set at *p*-value (*p* ≤ 0.05). Bonferroni correction was performed for linear mixed regression models to adjust for multiple testing. Statistical analyses were performed and figures were prepared using the R version 3.4.3 [28] and GraphPad prism version 6.05 for Windows (GraphPad Software, La Jolla, CA, USA).

## 3. Results

### 3.1. Characteristics of the Study Population

Baseline characteristics of the study population are provided in Table 1. Out of the 1291 individuals, 46% were never smokers, 37% were ex smokers, and 17% were current smokers. Smoking cessation was 18.6 years for ex smokers when calculating the mean value. Current smokers differed from never smokers in terms of sex (8% women compared to 27%) and hypertension (6% compared to 23%).

### 3.2. Smoking Increases GPR15 mRNA Expression

**Figure 1:**
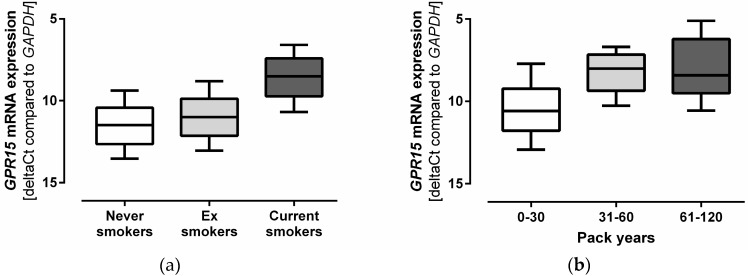
Smoking increases G protein-coupled receptor 15 (*GPR15*) mRNA expression. (**a**) *GPR15* mRNA expression is depicted for never smokers, ex smokers, and current smokers. It was significantly associated with current smoking (beta (ß) = −2.699, *p*-value (*p*) = 1.02 × 10^−77^). *n* = 191–542; (**b**) *GPR15* mRNA expression levels depending on cumulative smoking exposure are plotted for current smokers and ex smokers combined. They correlated significantly with the number of pack years smoked (ß = –0.0631, *p* = 5.95 × 10^−34^). One pack equals smoking 20 cigarettes per day for one year. *n* = 6-509. The linear mixed regression model was adjusted for age and sex. Box plot whiskers represent 10–90th percentile. *GPR15* mRNA expression is depicted as ΔCt values normalized to *GAPDH* mRNA expression. Lower ΔCt values indicate higher *GPR15* mRNA expression.

Gene expression was measured to determine the effect of smoking status on *GPR15* expression. *GPR15* mRNA expression strongly correlated with current smoking (beta (ß) = −2.699, *p* = 1.02 × 10^−77^) and was 7.6-fold higher in current smokers when compared to never smokers (Figure 1a). Furthermore, the effect of cumulative smoking exposure as indicated by the number of pack years significantly correlated with *GPR15* mRNA expression levels (ß = −0.0631, *p* = 5.95 × 10^−34^) (Figure 1b).

### 3.3. Smoking Cessation Alters GPR15 Expression 

**Figure 2:**
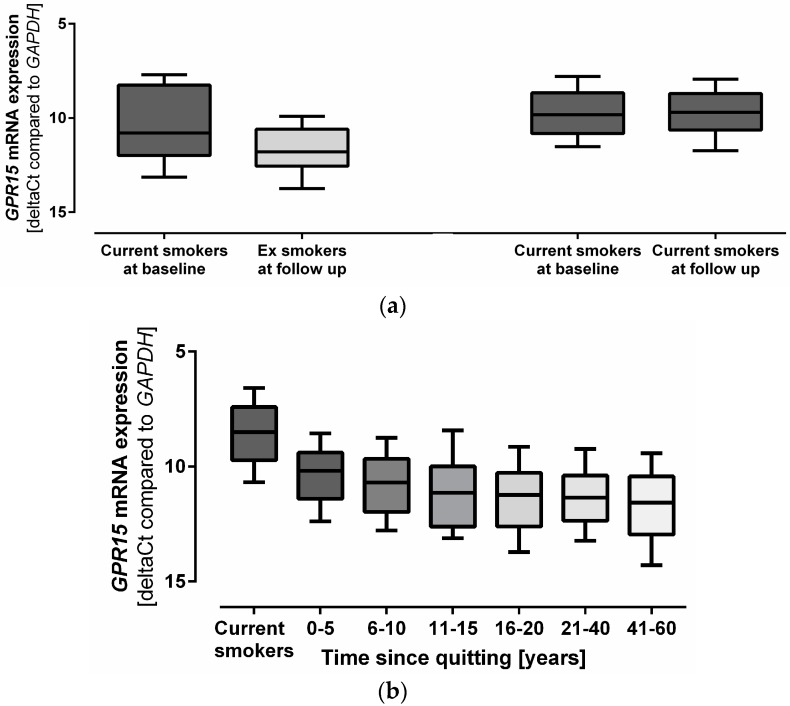
Effect of smoking cessation on G protein-coupled receptor 15 (*GPR15*) mRNA expression. (**a**) *GPR15* mRNA expression was compared between baseline (BL) and the five-years follow up (FU) visit. Quitting smoking significantly decreased *GPR15* mRNA expression about 19% (*p*) = 9.65 × 10^−5^). *n* = 39, paired *t*-test. (**b**) Time course of *GPR15* mRNA expression after smoking cessation. Time since smoking cessation significantly correlated with *GPR15* mRNA expression (ß = 0.031, *p* = 3.81 × 10^−6^). *n* = 20–186, linear mixed regression model adjusted for age and sex. Box plot whiskers represent 10–90th percentile. *GPR15* mRNA expression is depicted as ΔCt values normalized to *GAPDH* mRNA expression. Lower ΔCt values indicate higher *GPR15* mRNA expression.

To determine the longitudinal effect of smoking behavior on *GPR15* expression, gene expression levels at baseline and after a follow up time of five years were compared. Smokers who quit smoking within five years (*n* = 39) showed a significant decrease in *GPR15* expression (ß = 1.182, *p* = 9.65 × 10^−5^) in Figure 2a, which indicated that the change in smoking behavior affected *GPR15* mRNA expression. Furthermore, the time since smoking cessation significantly correlated with *GPR15* mRNA expression (ß = 0.031, *p* = 3.81 × 10^−6^) (Figure 2b), which shows that, within the first years after smoking cessation, *GPR15* mRNA expression decreased more rapidly (Figure S2).

### 3.4. Novel GPR15 DNA Methylation Sites Associated with Smoking

In previous studies, only the *GPR15* CpG3.98251294 site had been described in relation to smoking and *GPR15* expression [7,8,9,10,11,13]. To enhance the understanding of the molecular mechanisms regulating *GPR15* expression and to identify new *GPR15* CpG sites in relation to smoking, DNA methylation of nine additional CpG sites within the *GPR15* gene was measured.

**Figure 3:**
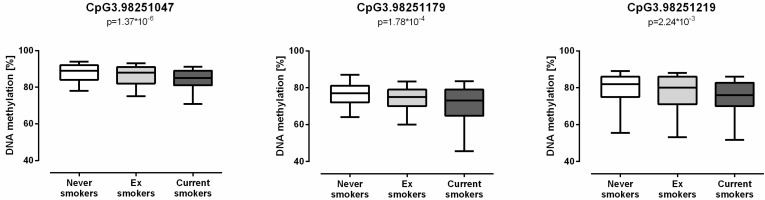
Decreased G protein-coupled receptor 15 (*GPR15*) DNA methylation in smokers. Methylation of *GPR15* DNA negatively correlated with smoking in the three newly identified sites CpG3.98251047, CpG3.98251179, and CpG3.98251219 (*n* = 114–582). Linear mixed regression model adjusted for age and sex with a significance threshold of 0.0083 after Bonferroni correction, the box plot whiskers represent 10–90th percentile.

Out of nine sites, DNA methylation of CpG3.98251047, CpG3.98251179, and CpG3.98251219 showed strong associations with smoking (ß = −3.376, *p* = 1.37 × 10^−6^; ß = −4.655, *p* = 1.78 × 10^−4^; and ß = −3.609, *p* = 2.24 × 10^−3^, respectively) (Figure 3). For sites CpG3.98251063, CpG3.98251070, and CpG3.98251294, no association with the smoking status was identified in our analyses.

**Figure 4:**
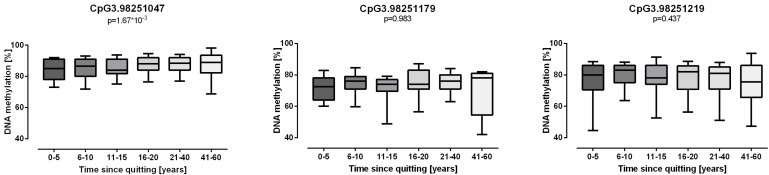
Methylation of G protein-coupled receptor 15 (*GPR15*) DNA slowly increased after smoking cessation. In ex smokers, *GPR15* DNA methylation of CpG3.98251047 gradually rose within years after quitting whereas CpG3.98251179 and CpG3.98251219 methylation was not associated with time since quitting (*n* = 9–153). Linear mixed regression model adjusted for age and sex with a significance threshold of 0.0083 after Bonferroni correction, Box plot whiskers represent 10–90th percentile.

As expected, following the decrease in *GPR15*, mRNA expression after smoking cessation (Figure 2) and *GPR15* DNA methylation at site CpG3.98251047 correlated with time since quitting in ex smokers (ß = 0.123, *p* = 1.67 × 10^−3^) (Figure 4), which showed increasing levels of *GPR15* methylation. For CpG sites CpG3.98251179 and CpG3.98251219, no significant change over time could be observed.

### 3.5. Decreased Levels of GPR15 DNA Methylation Correlate with Increased GPR15 mRNA Expression 

**Figure 5:**
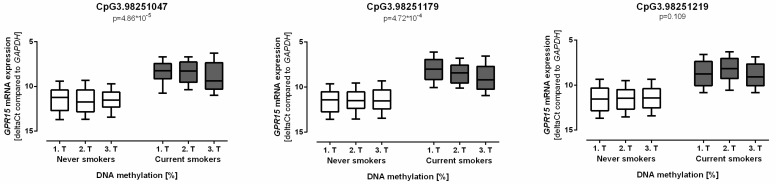
Methylation of G protein-coupled receptor 15 (*GPR15*) DNA was associated with *GPR15* mRNA expression. DNA methylation of CpG3.98251047 and CpG3.98251179 significantly correlated with *GPR15* mRNA expression. DNA methylation were divided in tertiles (T) to distinguish low, medium, and high DNA methylation levels. Subjects with lower DNA methylation had higher *GPR15* mRNA expression levels. *n* = 32–164, linear mixed regression model adjusted for age and sex with a significance threshold of 0.0083 after Bonferroni correction, Box plot whiskers represent 10–90th percentile. *GPR15* mRNA expression is depicted as ΔCt values normalized to *GAPDH* mRNA expression. Lower ΔCt values indicate higher *GPR15* mRNA expression.

Our data showed decreased levels of novel *GPR15* DNA methylation as well as increased levels of *GPR15* mRNA expression in smokers. Consequently, *GPR15* mRNA expression significantly correlated with *GPR15* DNA methylation at sites CpG3.98251047 (ß = 0.036, *p* = 4.86 × 10^−5^) and CpG3.98251179 (ß = 0.024, *p* = 4.72 × 10^−4^) (Figure 5). Site CpG3.98251219 showed a similar pattern. However, it did not reach statistical significance. In contrast to never smokers, CpG3.98251179 methylation was significantly correlated to *GPR15* mRNA expression in current smokers (ß = 0.032, *p* = 6.30 × 10^−3^).

## 4. Discussion

The association between smoking and multiple life-threatening diseases such as cardiovascular diseases has been well-known [1]. This is partly caused by the promotion of inflammatory processes [29]. In the present study, we investigated the effects of smoking on DNA methylation and mRNA expression of the *GPR15* locus in the population-based Gutenberg Health Study. Our results are manifold: (i) we identified associations between DNA methylation and smoking status in three previously unstudied CpG sites and (ii) showed that smokers had increased levels of *GPR15* expression, which slowly decreased after smoking cessation. In addition, (iii) smokers had decreased methylation levels of the three novel CpG sites within the *GPR15* gene and (iv) DNA methylation of the novel methylation sites negatively correlated with *GPR15* expression.

The *GPR15* gene comprises 15 CpG sites in total. However, up to now, only CpG3.98251294 (cg19859270) has been investigated [7,8,9,13]. Previous studies showed decreased CpG3.98251294 methylation in smokers compared to never smokers, which was associated with cumulative smoking exposure [7,8,9,30,31]. In this study, we discovered three novel methylation sites in relation to smoking: CpG3.98251047, CpG3.98251179, and CpG3.98251219, with CpG3.98251047 being the most strongly influenced by smoking. The three novel CpG sites presented an even stronger difference in DNA methylation of 4% to 6% between smokers and never smokers. Contrary to CpG3.98251294, methylation of none of the three new CpG sites was significantly associated with cumulative smoking exposure (Table S2).

Beside DNA methylation, mRNA expression of *GPR15* had been described to be influenced by smoking status [10,11,32,33]. Consistently, we showed that current smokers had 7.6-fold higher *GPR15* mRNA expression levels compared to never smokers. *GPR15* mRNA levels slowly decreased after smoking cessation. Additionally, our data showed that *GPR15* mRNA expression correlated with the number of pack years. Our results implicate that *GPR15* expression depends on the amount of cigarettes smoked per day and the duration of smoking.

Analyzing five years longitudinal gene expression data, we examined whether smoking changes *GPR15* mRNA expression. *GPR15* mRNA expression decreased after smoking cessation. Consistently, *GPR15* mRNA expression decreased with increasing time since quitting, which is in line with current knowledge [7]. Furthermore, our data showed that decreasing methylation levels of CpG3.98251047 and CpG3.98251179 were linked to increasing levels of *GPR15* mRNA, which indicated a direct influence of methylation status on *GPR15* gene expression. Even though the methylation sites analyzed in our study are not directly located in the *GPR15* promoter region but are within the single exon of *GPR15* and since *GPR15* consists of only 1252 bp, DNA methylation in close proximity to the promoter can influence transcription by inhibiting transcription factor binding [34]. Hence, as a potential mechanism, smoking might decrease *GPR15* DNA methylation in the progenitor cells, which could lead to an increase in GPR15 positive cells in the blood, which results in increased *GPR15* mRNA expression. 

Taken together, our data indicate that smoking decreases *GPR15* DNA methylation, which, in turn, leads to increased *GPR15* mRNA expression. Thereby, *GPR15* DNA methylation or *GPR15* mRNA expression might have a potential to act as new biomarkers for smoking behavior since factors such as second-hand smoke exposure, irregular smoking behavior, electrical cigarettes, and smokeless tobacco are almost impossible to estimate accurately by questionnaire.

The strengths of the presented work are the measurement of previously undescribed CpG methylation sites within the *GPR15* gene and its integration with mRNA expression. Our results originate from a large sample size with individuals from a population-based cohort and include longitudinal data. In this study, RNA was only available from enriched monocytes and DNA was available from leukocytes. *GPR15* RNA expression levels were low and the detected increase in *GPR15* expression in smokers could result from an increase in the number of GPR15 positive cells in the negatively selected monocyte fraction, as suggested by Bauer et al., rather than an upregulation of the gene expression [33]. The measurement of limited numbers of CpG sites, however, is a limitation of this study. Out of 15 CpG sites within *GPR15*, six sites could be analyzed due to technical limitations in the EpiTYPER amplicon design. Three sites were associated with smoking. Therefore, we cannot exclude the possibility that more methylation sites within the *GPR15* gene are present that are also associated with smoking. Contrary to previous results from microarray data [7,8,9,30,31], the CpG3.98251294 DNA methylation site was neither significantly associated with smoking nor *GPR15* expression in our analyses possibly because the EpiTYPER assay is less sensitive [26]. However, microarrays only include a limited selection of CpG sites. Using the EpiTYPER assay, we not only identified three novel CpG sites in relation to smoking, but the alterations in DNA methylation for these sites were even higher compared to Cpg3.98251294 [7,8,9,30,31]. Kõks et al. also determined DNA methylation of *GPR15* with the EpiTYPER assay. Comparably to our results, a different methylation between smokers and non-smokers was shown for CpG3.98251179 but not for CpG3.98251294 [10].

In summary, we identified three novel methylation sites within the *GPR15* gene whose methylation was affected by smoking, which led to an altered *GPR15* gene expression. These smoking-related changes in *GPR15* methylation and expression could perturb immune function and increase the risk for complex diseases with inflammatory pathogenesis, which could contribute to cardiovascular disease.

## Figures and Tables

**Table 1:** Characteristics of study individuals.

Characteristic	All	Never Smokers	Ex Smokers	Current Smokers
*n* (%)	1291	593 (46)	477 (37)	221 (17)
Females, *n* (%)	643 (50)	354 (27)	187 (15)	102 (8)
Age, years	55 (46–64)	57 (45–66)	57 (48–65)	50 (45–57)
Time since quitting, years	18 (7–28)	NA	18 (7–28)	NA
Pack years	0.1 (0.0–3.6)	NA	1.6 (0.6–3.7)	23.1 (11.7–36.0)

Continuous variables are described by median values (25th percentile to 75th percentile). Dichotomous variables are presented as total numbers (%). NA = data not available.

## Supplementary Materials

The following are available online at http://www.mdpi.com/2218-273X/8/3/74/s1. Table S1: Mass fragments detected by the EpiTYPER assay, Table S2: Results from linear mixed regression analyses, Figure S1: The *GPR15* locus in the UCSC Genome Browser human GRCh37/hg19 assembly, Figure S2: *GPR15* mRNA expression after smoking cessation. Availability of primary data is restricted due to limitations in ethical approvals.
