# Supplementary material for: Novel DNA Methylation Sites Influence GPR15 Expression in Relation to Smoking
Source: Biomolecules. 2018 Aug 20;8(3):74. doi: 10.3390/biom8030074 (PMC6163736; doi:10.3390/biom8030074)
Supplement: Supplementary file 1 [file biomolecules-08-00074-s001.zip › Supplements_revision1_trackchanges.pdf]

Supplementary material

Novel DNA methylation sites influence *GPR15* expression in relation to smoking

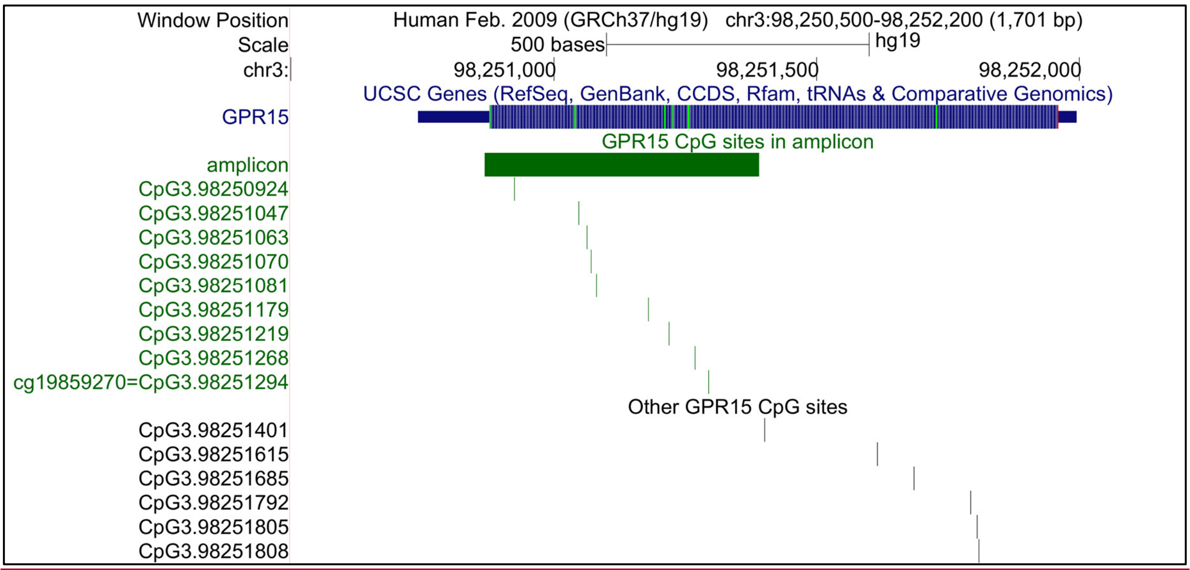

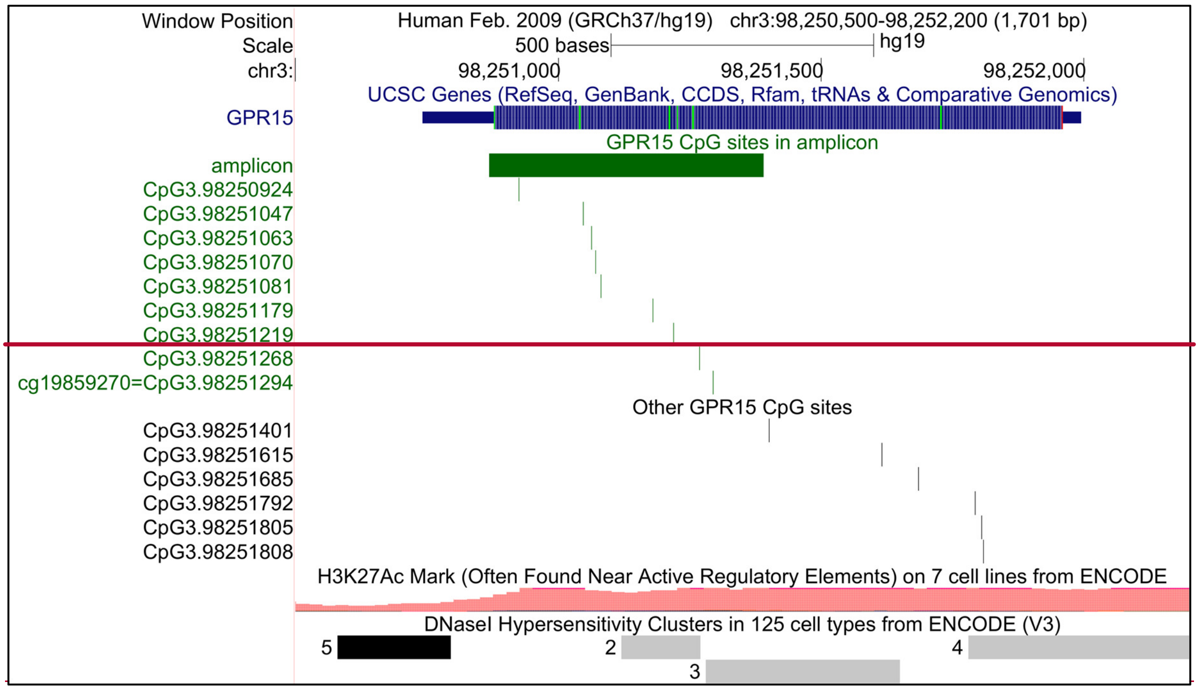

Figure S1. The *GPR15* locus in the UCSC Genome Browser human GRCh37/hg19 assembly. Nine out of 15 CpG sites within the *GPR15* exon were measured with the EpiTYPER amplicon. Due to low mass of the cleavage product, CpG3.98250924 and CpG3.98251081 could not be analysed. CpG3.98251268 was fully methylated in 90% of the samples. CpG3.98251047, CpG3.98251063, CpG3.98251070, CpG3.98251179, CpG3.98251219 and CpG3.98251294 were analysed. ~~Experimentally determined DNase hypersensitivity sites imply open chromatin and H3K27Ac peaks point to regulatory elements.~~ Adapted from http://genome.ucsc.edu/

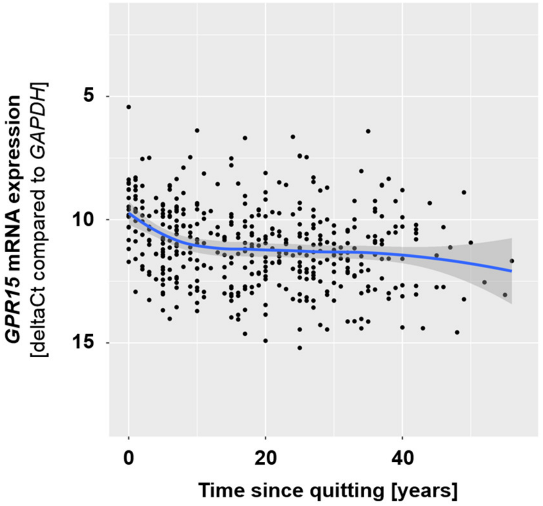

Figure S2. *GPR15* mRNA expression after smoking cessation. *GPR15* mRNA expression decreased more rapidly within the first years after smoking cessation. n = 427, *GPR15* mRNA expression is depicted as deltaCt values normalized to *GAPDH* mRNA expression. Lower deltaCt values indicate higher *GPR15* mRNA expression.

Table S1: Mass fragments detected by the EpiTYPER assay.

**DNA methylation site**	**Comment**	**Explanation**
CpG3.98250924	L_mass SN4	CpG unit with low mass (<1500Da) leading to unreliable methylation values; silent peak overlap with four non-methylated peaks
CpG3.98251047	NA	-
CpG3:98251063	SN1	silent peak overlap with one non-methylated peak
CpG3:98251070	SN1	silent peak overlap with one non-methylated peak
CpG3:98251081	L_mass SN3	CpG unit with low mass (<1500Da) leading to unreliable methylation values; silent peak overlap with three non-methylated peaks
CpG3.98251179	NA	-
CpG3.98251219	SN1	silent peak overlap with one non-methylated peak
CpG3.98251268	SN2	silent peak overlap with two non-methylated peaks
CpG3:98251294	SN1	silent peak overlap with one non-methylated peak

Table S12: Results from linear mixed regression analyses.

**Dependent variable**	**Independent variables**	**Effect**	**Standard error**	**p value**
GPR15mRNA	Age + Sex + Current_smoking	-2.699	0.134	1.02e-77
GPR15mRNA	Age + Sex + Pack_years	-0.0631	0.005	2.95e-34
GPR15mRNA	Age + Sex + Time_since_quitting	0.0310	0.007	3.81e-06

CpG3.98251047	Age + Sex + Current_smoking	-3.376	0.694	1.37e-06
CpG3:98251063	Age + Sex + Current_smoking	-1.495	1.951	0.443
CpG3:98251070	Age + Sex + Current_smoking	-4.607	3.219	0.153
CpG3.98251179	Age + Sex + Current_smoking	-4.655	1.234	1.78e-04
CpG3.98251219	Age + Sex + Current_smoking	-3.609	1.178	2.24e-03
CpG3:98251294	Age + Sex + Current_smoking	2.439	2.336	0.297
CpG3.98251047	Age + Sex + Pack_years	-0.030	0.041	0.461
CpG3:98251063	Age + Sex + Pack_years	-0.045	0.103	0.661
CpG3:98251070	Age + Sex + Pack_years	-0.181	0.165	0.279
CpG3.98251179	Age + Sex + Pack_years	-0.065	0.078	0.406
CpG3.98251219	Age + Sex + Pack_years	0.021	0.066	0.755
CpG3:98251294	Age + Sex + Pack_years	0.008	0.123	0.950
CpG3.98251047	Age + Sex + Time_since_quitting	0.123	0.039	1.67-03
CpG3:98251063	Age + Sex + Time_since_quitting	-0.040	0.102	0.695
CpG3:98251070	Age + Sex + Time_since_quitting	-0.014	0.201	0.945
CpG3.98251179	Age + Sex + Time_since_quitting	0.001	0.068	0.983
CpG3.98251219	Age + Sex + Time_since_quitting	-0.049	0.063	0.437
CpG3:98251294	Age + Sex + Time_since_quitting	-0.032	0.128	0.802
GPR15mRNA	Age + Sex + CpG3.98251047	0.036	0.009	4.86e-05
GPR15mRNA	Age + Sex + CpG3:98251063	0.000	0.002	0.827
GPR15mRNA	Age + Sex + CpG3:98251070	0.005	0.004	0.24
GPR15mRNA	Age + Sex + CpG3.98251179	0.024	0.007	4.72-04
GPR15mRNA	Age + Sex + CpG3.98251219	0.006	0.004	0.109
GPR15mRNA	Age + Sex + CpG3:98251294	-0.002	0.002	0.407
